# Emergence of multidrug-resistant *Providencia rettgeri* clone in food-producing animals: A public health threat

**DOI:** 10.1016/j.onehlt.2024.100887

**Published:** 2024-09-03

**Authors:** Tiago Barcelos Valiatti, Fernanda Fernandes Santos, Francisco Ozório Bessa-Neto, Ruanita Veiga, Simone Simionatto, Gleyce Hellen de Almeida Souza, Márcia Soares Mattos Vaz, Antônio Carlos Campos Pignatari, Rodrigo Cayô, Ana Cristina Gales, Guarani Network

**Affiliations:** a Universidade Federal de São Paulo (UNIFESP), Laboratório Alerta, Division of Infectious Diseases, Department of Internal Medicine, Escola Paulista de Medicina (EPM), São Paulo, SP, Brazil; b Universidade Federal de São Paulo (UNIFESP), Laboratório de Imunologia e Microbiologia (LIB), Setor de Biologia Molecular, Microbiologia e Imunologia, Departamento de Ciências Biológicas (DCB), Instituto de Ciências Ambientais, Químicas e Farmacêuticas (ICAQF), Diadema, SP, Brazil; c Universidade Federal da Grande Dourados (UFGD), Laboratório de Pesquisa em Ciências da Saúde, Dourados, MS, Brazil; d Universidade Federal de São Paulo (UNIFESP), Laboratório Especial de Microbiologia Clínica (LEMC), Division of Infectious Diseases, Department of Internal Medicine, Escola Paulista de Medicina (EPM), São Paulo, SP, Brazil

**Keywords:** Genomic surveillance, Gram-negative bacilli, Enterobacterales, One Health, Resistome, Carbapenemases

## Abstract

The occurrence of carbapenemases encoding genes in *Providencia rettgeri* is a critical public health concern since this species has intrinsic resistance to several antimicrobials, including polymyxins. The identification of this multidrug-resistant (MDR) pathogen outside the hospital setting has become increasingly frequent, and raises an alert for the global health agencies, as they indicate a possible spread of such pathogens. Herein, we described three MDR *P. rettgeri* isolates carrying a diversity of antimicrobial resistance genes (ARGs) isolated from stool samples of swine and bovine in Brazil. Molecular analysis revealed that all isolates belonged to the same clone. The whole genome sequencing (WGS) of a representative isolate (PVR-188) was performed by MiSeq Illumina® platform, while the assembling and annotation was achieved using SPAdes and Prooka, respectively. The WGS analyses indicated the presence of ARGs that confer resistance to β-lactams (*bla*_NDM-1_, *bla*_CTX-M-2_), quinolones (*qnrD1*), aminoglycosides (*aadA*2, *aadA1*, *aph(3′)-Via*), phenicol (*catB2*), sulfonamides (*sul1*, *sul2*), and trimethoprim (*dfrA12*, *dfrA1*). The presence of three plasmid replicons (Col3M, IncQ1, and IncT) was detected, but no phage sequences were found. The phylogenetic analyses confirmed the genomic relationship of the PVR-188 with *P. rettgeri* isolates recovered from animals and humans in the USA and Malaysia. In conclusion, we report the occurrence of MDR *P. rettgeri* clone colonizing the gut microbiota of food-producing animals in Brazil, revealing the spread of this pathogen beyond hospital boundaries.

*Providencia rettgeri* is a Gram-negative bacillus (GNB) belonging to the *Enterobacteriales* order and *Morganellaceae* family [1,2]. Although this enterobacteria is considered an opportunistic pathogen, being mainly associated with urinary tract infections [3], it can also be an etiologic agent of other infections, such as bacteremia [4], traveler's diarrhea [5,6], and eye infections [7]. In recent years, reports of multidrug-resistant (MDR) *P. rettgeri* strains have emerged worldwide In most cases, this phenomenon has been attributed to the acquisition of the *bla*_NDM-1_ [1,[8], [9], [10], [11], [12], [13], [14]].

The production of carbapenemases by *P. rettgeri* is worrisome due to its intrinsic resistance to penicillins, first-generation cephalosporins, tetracyclines, nitrofurantoin, and polymyxins [9]. To date, most studies reporting the occurrence of carbapenemase-producing *P. rettgeri* have been limited to clinical strains, and this phenomenon is rarely described outside the hospital environment [1,[8], [9], [10], [11], [12], [13], [14]]. It is noteworthy that, in recent years, it has become clear that the dissemination of AMR involves a multifactorial chain within the context of a one health, thus demonstrating the importance of carrying out surveillance studies that include environmental and animal samples, to assist in understanding the spread of these multidrug-resistant strains that are impacting the clinical environment.

Herein, we describe the occurrence of MDR *P. rettgeri* isolates recovered from the gut microbiota of food-producing animals in Brazil. The isolates PVR-188, PVR-191, and PVR-231 were recovered during a national antimicrobial resistance surveillance study at the human-animal interface carried out in 2020. The *P. rettgeri* isolates were recovered from stool samples of swine (*n* = 2) and cattle (*n* = 1) from two rural properties nearby the city of Dourados, located in the Mato Grosso do Sul state, Midwestern Brazilian Region.

All *P. rettgeri* isolates were recovered from CHROMagar™ Orientation (Becton Dickson and Company™, New Jersey, USA) plates supplemented with 2 μg/mL of meropenem (Sigma-Aldrich, St. Louis, USA) and incubated under aerobiosis at 35 ± 2 °C overnight. The identification at species level of the *P. rettgeri* isolates as was confirmed by MALDI-TOF MS using Microflex LT spectrometry and MALDI Biotyper *vs.* 3.3 software (Bruker Daltonics, Massachusetts, USA), according to manufactor's recommendations. Minimum inhibitory concentrations (MIC) were determined by agar dilution for the following antimicrobials (Sigma-Aldrich, St. Louis, USA): aztreonam, ceftriaxone, ceftazidime, cefepime, ertapenem, imipenem, meropenem, gentamicin, amikacin, ciprofloxacin, and levofloxacin (Sigma San Luis, Missouri, USA). The susceptibility results were interpreted according to the Brazilian Antimicrobial Susceptibility Testing Committee (BrCAST)/EUCAST guidelines (https://brcast.org.br/). High resistance rates were observed for the 11 antimicrobials tested against the *P. rettgeri* isolates: aztreonam (MICs, ≥128 μg/mL), ceftazidime (MICs, ≥ 256 μg/mL), ceftriaxone (MICs 128–256 μg/mL), cefepime (MICs 64–128 μg/mL), ertapenem (MICs 128 - >256 μg/mL), imipenem (MICs 64–128 μg/mL), meropenem (MICs 128–256 μg/mL), amikacin (MICs 128–256 μg/mL), gentamicin (MICs 32–64 μg/mL) ciprofloxacin (MICs, >64 μg/mL), and levofloxacin (MICs, 64 μg/mL).

The screening for β-lactamase-encoding genes (*bla*_TEM_-like*, bla*_SHV_-like*, bla*_CTX-M_-like*, bla*_GES_-like, *bla*_KPC_-like, *bla*_NDM_-like, *bla*_IMP_-like, *bla*_VIM_-like, *bla*_SPM_-like, *bla*_GIM_-like, *bla*_SIM_-like, *bla*_OXA-48_-like) was performed by PCR followed by DNA sequencing using specific primers (Thermo Fisher Scientific™, Delaware, EUA), as previously published [15,16]. Molecular analysis demonstrated that all *P. rettgeri* isolates carried the *bla*_CTX-M-2_ and *bla*_NDM-1_ genes. These results corroborate previous studies carried out worldwide, which demonstrated that resistance to carbapenems in *P. rettgeri* was strongly associated with the production of NDM-1 [1,[8], [9], [10], [11], [12], [13], [14]]. Interestingly, the first description of *bla*_NDM-1_ in South America occurred in a *P. rettgeri* clinical isolate recovered from a Uruguayan Hospital in 2012 [17]. One year later, a NDM-1-producing *P. rettgeri* isolate was reported in Brazil [18]. Since then, the spread of *bla*_NDM-1_ in other clinically relevant GNB species has been observed in different Brazilian geographic regions [[19], [20], [21]].

The genetic similarity of *P. rettgeri* isolates was determined by Pulsed Field Gel Electrophoresis (PFGE) using the CHEF-DR II system (Bio-Rad Laboratories, California, USA) and the restriction enzyme *SfiI* (New England Biolabs, Ipswich, UK). Since the three *P. rettgeri* isolates showed identical PFGE patterns, one representative isolate (PVR-188) was randomly chosen for Whole Genome Sequencing (WGS). For this purpose, total bacterial DNA was extracted using the QIAamp DNA Mini kit (Qiagen, Hilden, Germany), following the manufacturer's recommendations. DNA libraries were prepared using the Nextera® XT kit (Illumina® Inc., San Diego, CA) and sequenced at MicrobesNG of University of Birmingham (UK) on the Illumina® HiSeq™ 2500 System using 2 × 250-bp paired-end mode platform. The WGS data obtained were assembled using SPAdes software version 3.9.1 [22] and annotation was performed using Prokka version 1.12 [23]. The genome assembly metric was calculated using QUAST (http://quast.sourceforge.net/). All software was used with default settings. Resistome analysis was performed using ResFinder 4.1 (https://cge.cbs.dtu.dk/services/ResFinder/) and plasmid replicons by PlasmidFinder 2.1 (https://cge.cbs.dtu.dk/services/PlasmidFinder/), both belonging to the Center for Genomic Epidemiology (CGE) platform (http://www.genomicepidemiology.org/). GyrA and ParC sequences of PR-188 were compared with the reference isolate *P. rettgeri* AR-0082 (GenBank: CP029736.1) using Clustal Omega software (https://www.ebi.ac.uk/Tools/msa/clustalo/). The presence of phages in the PVR188genome was investigated by using PHASTER web server (https://phaster.ca/). A variety of antimicrobial resistance genes (ARGs) were observed, in addition to *bla*_NDM-1_ and *bla*_CTX-M-2_, in the genome of PVR-188 isolate, as follows: fluoroquinolones (*qnrD1*), aminoglycosides (*aadA2*, *aadA1*, *aph(3′)-Via*), phenicol (*catB2*), sulfonamides (*sul1*, s*ul2*), and trimethoprim (*dfrA12*, *dfrA1*). Additionally, we identified chromosomal mutations in the *gyrA* (F50Y, S83I, E480D, D678E, I852N, N854I) and *parC* (S158N, Y219F, S471A, T585S, D749E, T750S) genes. Furthermore, the plasmid replicons Col3M, IncQ1, and IncT were also detected. However, no phage sequences were found in its genome. The *bla*_NDM-1_ gene was within a 6861 bp-sized contig that showed 100 % identity and 98 % coverage with larger plasmids from several GNB species, such as: *Escherichia coli* (GI: LR697127.1), *Klebsiella pneumoniae* (GI: CP021962.1), *Citrobacter freundii* (KP770032.1), *P. rettgeri* (GI: KF295828.1), *Acinetobacter cumulans* (GI: CP035935.1), *Acinetobacter bereziniae* (GI: KP282691.1), *Acinetobacter nosocomialis* (GI: CP010370.2), and *Acinetobacter lwoffii* (GI: JQ060896.1). Due to the short-read sequencing methodology employed by our study, we were not able to assemble the entire genetic context of the *bla*_NDM-1_ gene. However, it could be observed that upstream *bla*_NDM-1_ in PVR-188 genome there was a copy of IS*Aba125*, corroborating with previous studies that reported the mobilization of *bla*_NDM-1_ associated with this insertion sequence in *Tn125* [[24], [25], [26]].

Phylogenetic analysis using the Similar Genome Finder tool of Pathosystems Resource Integration Center (PATRIC) platform (https://www.patricbrc.org/) demonstrated that the PVR-188 was related with 28 other *P. rettgeri* genomes deposited at GenBank®/NCBI isolates. A SNP-based phylogenetic tree using the reference *P. rettgeri* ATCC® 29944™ genome was constructed using the CSI Phylogeny v.1.4 tool (https://cge.cbs.dtu.dk/). In addition, the visualization and annotation of the SNP-based tree were performed with iTol v.6 (https://itol.embl.de/). The SNPs values between PVR-188 isolate and others *P. rettgeri* isolates recovered from human and animal sources worldwide varied from 226 to 11,781. Additionally, the PVR-188 isolate was grouped into a clade (Fig. 1) with three other *P. rettgeri* clinical isolates isolated in the United States [GCA_018068585.1 (5,338) and GCA_015739425.1 (226 SNPs)] and Malaysia [GCA_004795525.1 (4,936 SNPs)]. Moreover, as verified in the PVR-188 isolate, most of *P. rettgeri* isolates included in the phylogenetic analysis carried *bla*_NDM-1_ or *bla*_NDM-7_, as well as a diversity of other ARGs (Fig. 2), showing the potential of this pathogen as a reservoir of ARGs to other GNBs.

**Fig. 1:**
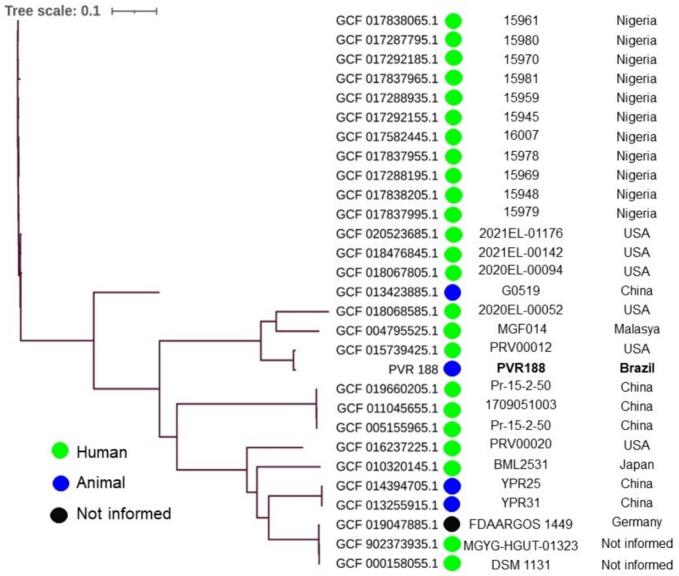
SNP-based phylogenetic tree analysis of PVR-188 isolate and 28other *P. rettgeri* genomes recovered from distinct hosts worldwide and deposited in the GenBank®/NCBI database until December 2023.

**Fig. 2:**
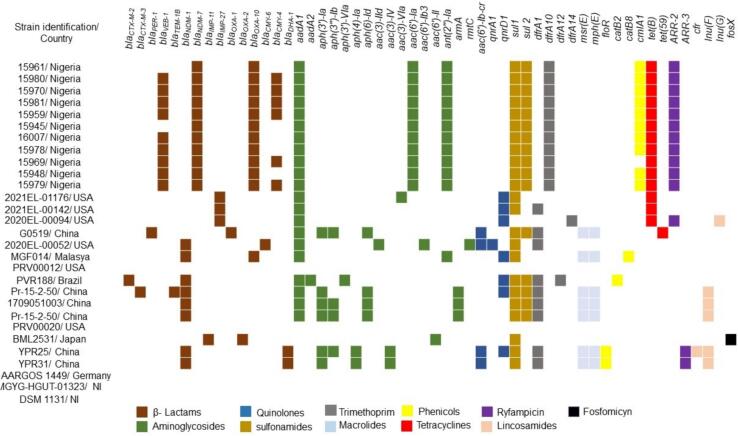
Heat map of the resistome of *P. rettgeri* isolates isolated from humans and animals from different geographic regions. NI: not informed.

Virtually, all currently available studies have reported MDR *Providencia spp.* isolates causing infections in humans. Herein, we have firstly shown to the best of our knowledge, the occurrence of MDR *P. rettgeri* isolates carrying a variety of ARGs colonizing the gut microbiota of food-producing animals in Latin America. This data is concerning, as the presence of these pathogens in animal feces may directly contribute to the spread and persistence of antimicrobial resistance genes (ARGs) in the environment. [[27], [28], [29]]. Besides, although it is difficult to ascertain the common origin of contamination by NDM-1-producing *P. rettgeri* clone in food-producing animals, we believe that the anthropic activities may have exerted a key role in this finding.

One possible limitation of the present study is that it examined a limited number of isolates from a specific region in Brazil. While the findings provide valuable insights, including more diverse samples from additional regions could further enhance our understanding of the spread and evolution of MDR *P. rettgeri* clones.

In conclusion, to the best of our knowledge, this is the first report of NDM-1-producing *P. rettgeri* in food-producing animals in Latin America, revealing that these animals can serve as significant reservoirs of MDR bacteria. Furthermore, the data presented here emphasize the importance of a One Health approach in surveillance studies, as the dissemination of antimicrobial resistance is interconnected across different ecological niches.

## Guarani Network

Regional University of Blumenau (FURB), Blumenau - SC, Brazil: Alessandro Conrado de Oliveira Silveira and Eleine Kuroki Anzai. Seção de Bacteriologia e Micologia, Instituto Evandro Chagas (IEC), Ananindeua - PA, Brazil: Cintya de Oliveira Souza, Danielle Murici Brasiliense, Márcia de Nazaré Miranda Bahia, William Alencar de Oliveira Lima. Postgraduate Program in Medical Microbiology, Group of Applied Medical Microbiology, Federal University of Ceará (UFC), Fortaleza - CE, Brazil: Débora de Souza Collares Maia Castelo-Branco and Glaucia Morgana de Melo Guedes. Laboratory of Molecular Biology of Microorganisms, University São Francisco (USF), Bragança Paulista - SP, Brazil: Lúcio Fábio Caldas Ferraz and Walter Aparecido Pimentel Monteiro. Universidade Federal de São Paulo (UNIFESP), Laboratório Especial de Microbiologia Clínica (LEMC), Division of Infectious Diseases, Department of Internal Medicine, Escola Paulista de Medicina (EPM), São Paulo - SP, Brazil: Carlos Roberto Vieira Kiffer.

## Ethical approval

Ethics approval for this study was obtained from the Research Ethics Committee SNP ranging 0 to 12.022– CEP (process numbers 3.116.383) and the Committee on Ethics in the Use of Animals - CEUA (process number 2607170119) of the Universidade Federal de São Paulo (UNIFESP). This project was also registered by the National System for the Management of Genetic Heritage and Associated Traditional Knowledge (process number: AA1668A).

## Funding

This study was supported by the National Council for Science and Technological Development - CNPq (process numbers 402659/2018-0 and 443805/2018-0) and by 10.13039/100000865Bill & Melinda Gates Foundation (process number: OPP1193112). Under the grant conditions of the Bill & Melinda Gates Foundation, a Creative Commons Attribution 4.0 Generic License has already been assigned to the Author Accepted Manuscript version that might arise from this submission.

## Accession number

The PVR-188 genome was deposited in the GenBank®/NCBI database under the accession number SAMN31228607, BioProject PRJNA888994.

## CRediT authorship contribution statement

**Tiago Barcelos Valiatti:** Writing – review & editing, Writing – original draft, Investigation, Formal analysis, Conceptualization. **Fernanda Fernandes Santos:** Writing – original draft, Investigation, Formal analysis, Conceptualization. **Francisco Ozório Bessa-Neto:** Methodology, Investigation. **Ruanita Veiga:** Methodology, Investigation. **Simone Simionatto:** Writing – review & editing, Supervision, Investigation, Conceptualization. **Gleyce Hellen de Almeida Souza:** Methodology, Investigation. **Márcia Soares Mattos Vaz:** Methodology, Investigation. **Antônio Carlos Campos Pignatari:** Writing – review & editing, Conceptualization. **Rodrigo Cayô:** Writing – review & editing, Writing – original draft, Conceptualization. **Ana Cristina Gales:** Writing – review & editing, Supervision, Investigation, Funding acquisition, Conceptualization.

## Declaration of competing interest

A.C.G. has recently received research funding and/or consultation fees from Aché, BioMerieux, Eurofarma, MSD, Pfizer, Sandoz, União Química, and United Medical. Other authors have nothing to declare. This study was not financially supported by any Diagnostic/Pharmaceutical company.

## Data Availability

The PVR-188 genome was deposited in the GenBank®/NCBI database under the accession number SAMN31228607, BioProject PRJNA888994.
